# Synthesis and Biological Evaluation of Chromenylurea and Chromanylurea Derivatives as Anti-TNF-α agents that Target the *p*38 MAPK Pathway

**DOI:** 10.3390/molecules19022004

**Published:** 2014-02-13

**Authors:** Xingzhou Li, Xinming Zhou, Jing Zhang, Lili Wang, Long Long, Zhibing Zheng, Song Li, Wu Zhong

**Affiliations:** Laboratory of Computer-Aided Drug Design & Discovery, Beijing Institute of Pharmacology and Toxicology, 27 Taiping Road, Beijing 100850, China; E-Mails: xingzhou1970@gmail.com (X.L.); chemistry20120720@gmail.com (X.Z.); rayzhangjing@sina.com (J.Z.); Wangll63@126.com (L.W.); long072811@126.com (L.L.); zzbcaptain@aliyun.com (Z.Z.); lis.lisong@gmail.com (S.L.)

**Keywords:** kinase inhibitor, *p*38 MAPK, 2*H*-chromenylurea, chromanyl-urea

## Abstract

A series of 1-aryl-3-(2*H*-chromen-5-yl)urea and 1-aryl-3-(chroman-5-yl)urea derivatives were designed, synthesized and evaluated for their inhibitory activities towards TNF-α production in lipopolysaccharide-stimulated THP-1 cells. The most active compound, **40g**, inhibited TNF-α release with an IC_50_ value of 0.033 μM, which is equipotent to that of BIRB796 (IC_50_ = 0.032 μM).

## 1. Introduction

The p38 mitogen-activated protein kinase (*p*38MAPK) plays a key role in inflammatory responses through the production of cytokines and inflammatory mediators such as TNF-α and IL-1β [1]. At least four distinct homologues, standardized in the nomenclature as p38α, β, γ and δ, have been identified. The inhibition of *p*38MAPKα is considered to be a promising therapeutic strategy for chronic inflammatory diseases such as rheumatoid arthritis [2], psoriasis, inflammatory bowel disease [2], and chronic obstructive pulmonary disease [3]. Recent studies have also revealed that *p*38MAPKα inhibitors may have therapeutic potential in the treatment of cancer [4,5], neuropathic pain [6] and periodontal diseases [7]. Consequently, considerable effort has been directed toward the development of *p*38MAPKα inhibitors as potential anti-inflammatory and anticancer drug.

**Figure 1:**
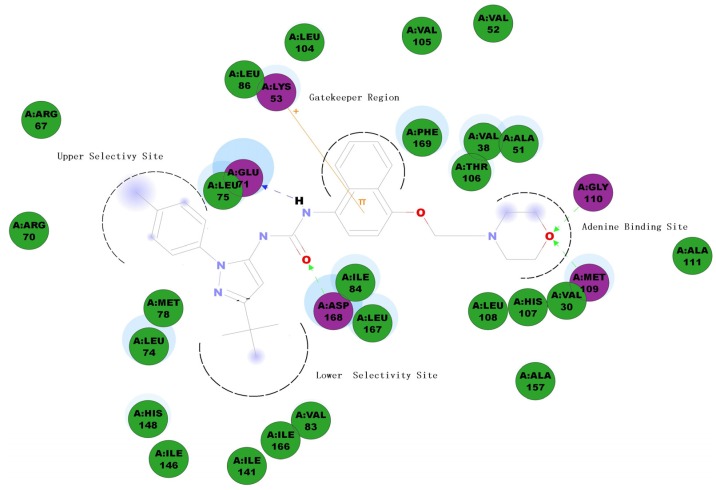
Structure of the compound BIRB796 and two-dimensional *p*38 MAPKα/BIRB796 interaction map.

*p*38MAPKα inhibitors, like many other kinase inhibitors, can be classified into two types based on their mode of action: ATP-competitive inhibitors, which bind to an ATP-binding site, and non-ATP-competitive or allosteric inhibitors. Allosteric inhibitors utilize the ATP binding cleft and a hydrophobic allosteric pocket created when the activation loop adopts the inactive “Asp-Phe-Gly (DFG)-out” conformation. Because allosteric inhibitors do not compete directly with ATP or substrate, they can offer a significant kinetic advantage over ATP competitive inhibitors. In addition, because the allosteric pocket is less conserved than the ATP binding region, allosteric inhibitors usually have better kinase selectivity profiles than ATP competitive inhibitors [8]. BIRB796 is a typical allosteric *p*38MAPKα inhibitor with an *N*-pyrazole-*N'*-naphthyl urea scaffold. The crystal structure of the *p*38MAPKα/BIRB796 complex shows that BIRB796 fits well into the DFG-out conformation by forming several tight interactions. (Figure 1 graphically depicts a two-dimensional *p*38MAPKα/BIRB796 interaction map.) By taking advantage of these interactions, BIRB796 achieves potent *p*38MAPKα inhibition with a Kd value of 0.1 nM [9]. The major drawback of BIRB796 is its hepatotoxicity [10], which may be caused by one of its metabolic intermediates, naphthalene epoxide [11]. We therefore performed scaffold modification and structure–activity relationship (SAR) investigations of BIRB796 and its analogues to discover novel *p*38MAPKα inhibitors with drug-like properties.

According to the crystal structure of the *p*38MAPKα/BIRB796 complex (PDB 1VK2) [9], the carbonyl from the urea linkage of BIRB796 accepts hydrogen bonds from the backbone NH of Asp168, while the NH from the urea linkage forms hydrogen bonds to the Glu71 side chain. Such a hydrogen bonding network is essential to maintain the *p*38MAPKα inhibition activity, so we preserved this urea linkage in the compounds we designed. The naphthyl ring of BIRB796 pushes very deep into the hydrophobic gatekeeper pocket. It is known that such a hydrophobic interaction is essential to obtain high activity and selectivity for a *p*38MAPKα inhibitor [12]*.* A pi-cation interaction has also been observed between the naphthyl ring and the cationic amino groups in the side chains of Lys53. To preserve the activity and selectivity, and to eliminate the hepatotoxicity caused by the naphthyl ring, we attempted to replace the naphthalene ring with two other aromatic hydrophobic scaffolds: 2*H*-chromene and chromane. In BIRB796, the morpholinoethoxy group occupies the adenine binding site, and forms hydrogen bond interactions with the residue of Met109 and Gly110. According to SAR information of kinase inhibitors, a variety of functional groups could be well tolerated by this adenine binding site. We therefore attempted to either replace this morpholinoethoxy moiety with a 2-morpholino-2-oxoethoxy moiety, or to replace the morpholine ring with a larger aliphatic group (2,6-dimethylmorpholine) or aromatic hydrogen bond-accepting moieties (triazole, pyridine or imidazole) with the aim of increasing the binding affinity. BIRB796 also utilizes a unique allosteric pocket created when the activation loop adopts the “DFG-out” conformation. This allosteric pocket can be divided into two selectivity sites: the lower selectivity site, occupied by the *t*-butyl group, and the upper selectivity site, occupied by the *p*-tolyl group. It has been reported that the *t*-butyl group fits well with the highly conserved lower selectivity site, while the upper selectivity site is less conserved and offers a unique position to enable *p*38MAPKα inhibitory activity and kinase selectivity [13]. On the basis of this knowledge, the *t*-butyl group was preserved in our compounds, while we attempted to substitute the 4-tolyl group with another substituted phenyl to investigate the SAR around the phenyl ring unit. It was also of interest to us that whether substituting the pyrazole ring with its isosteres, such as oxazole and imidazole, would increase the inhibitory activity for *p*38MAPKα. On the basis of the above information, a series of *N*-aryl-*N'*-chromenyl urea and *N*-aryl-*N'*-chromanyl urea derivatives was designed, synthesized and evaluated for their inhibitory activity against TNF-α release.

## 2. Results and Discussion

**Scheme 1:**
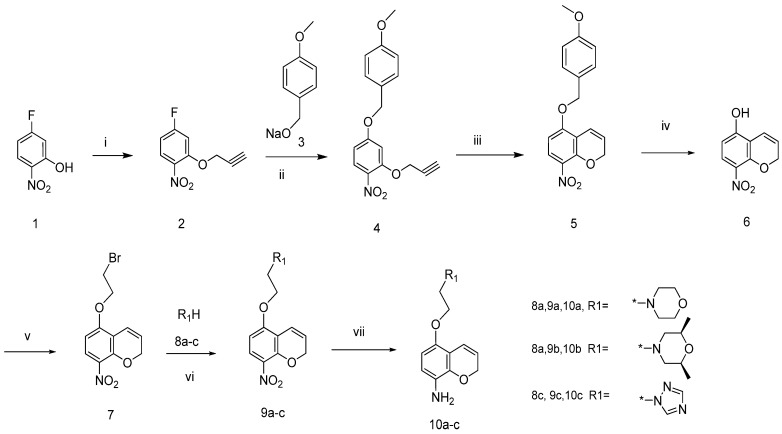
Synthesis of 5-alkoxy-2*H*-chromen-8-amines.

**Scheme 2:**
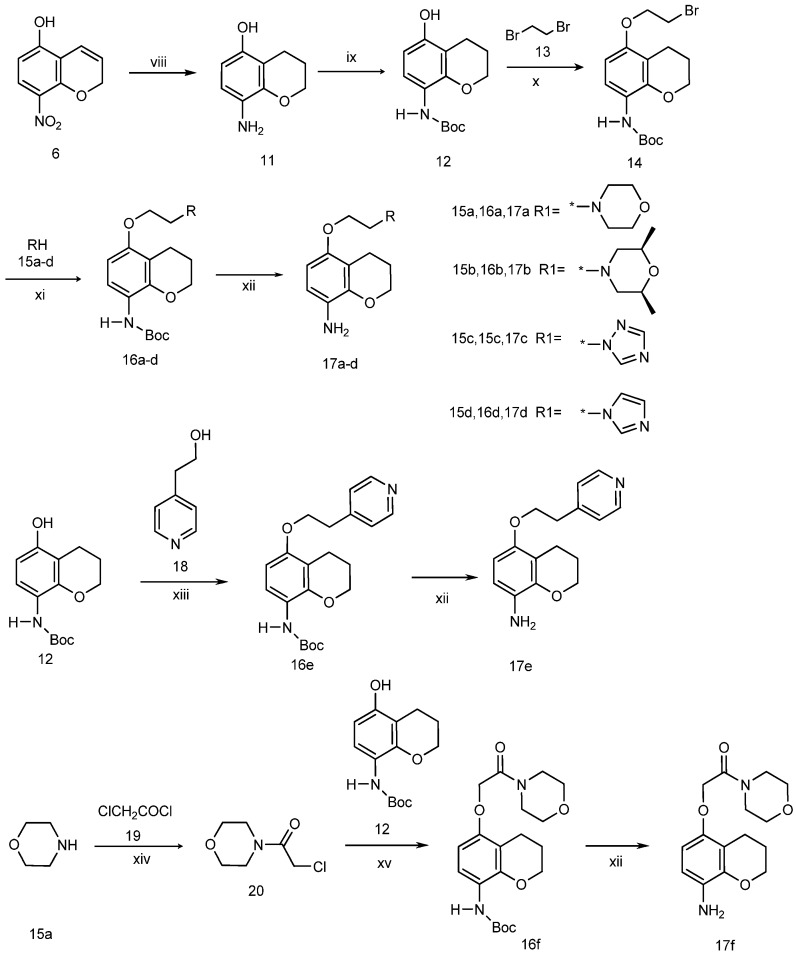
Synthesis of 5-alkoxychroman-8-amines.

**Scheme 3:**
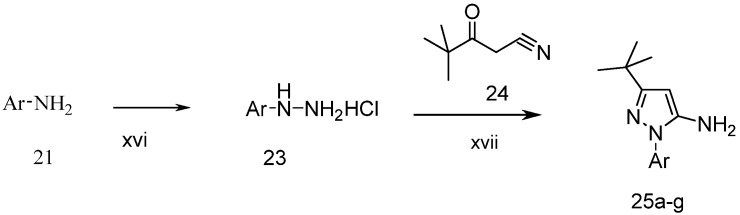
Synthesis of 1-aryl-3-*tert*-butyl-*1*H-pyrazol-5-amines.

**Scheme 4:**
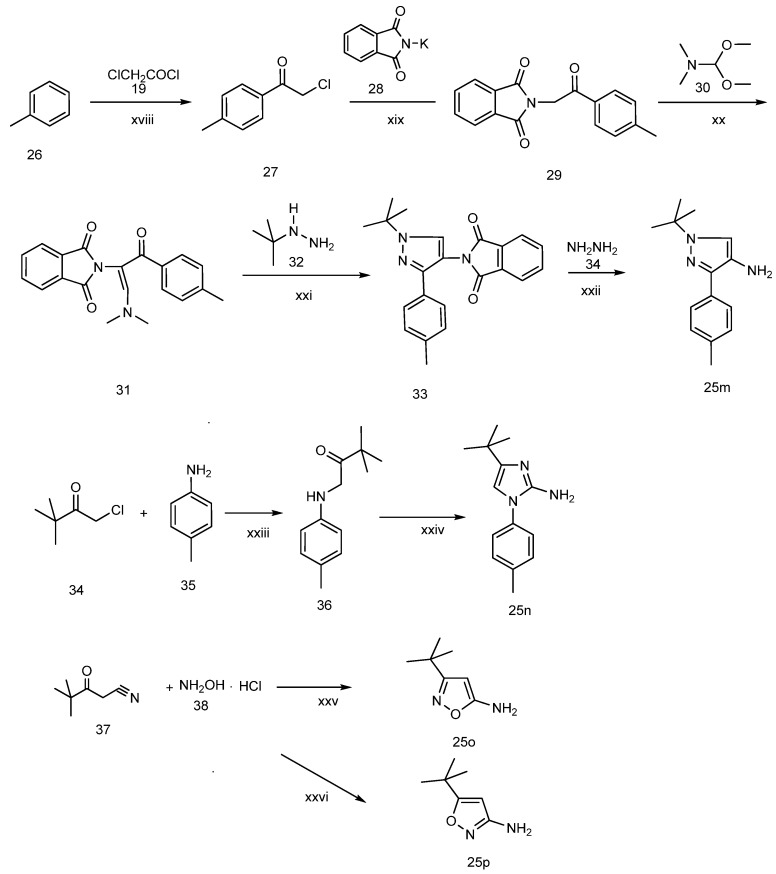
The synthesis of arylamines **25m**, **25n**, **25o**, **25p**.

**Scheme 5:**
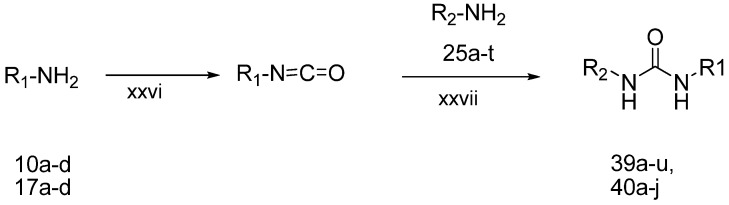
The synthesis of *N*-aryl-*N'*-chromenylurea and *N*-aryl-*N'*-chromanylurea derivatives.

To obtain our target compounds, two series of aromatic amines [one series of 5-alkoxy-2*H*-chromen-8-amines (Scheme 1) and 5-alkoxychroman-8-amines (Scheme 2) and another series containing pyrazol-5-amines (Scheme 3) and their isosteres (Scheme 4)] were first prepared, and then the target urea derivatives obtained by coupling these two series of aromatic amines. The key to the success of this synthetic strategy was to prepare the 5-alkoxychroman-8-amines and 5-alkoxy-2*H*-chromen-8-amines efficiently. A simple procedure for the synthesis of 5-morpholinoethoxy-2*H*-chromen-8-amines, starting from commercially available 2-nitro-5-fluorophenol, had been developed by our group [13]. In this paper, an improved and more diverse procedure, using 5-hydroxy-8-nitro-2*H*-chromene as a key intermediate, was developed and executed to prepare the desired 5-alkoxy-2*H*-chromen-8-amines and 5-alkoxychroman-8-amines.

The general synthetic approach to the 5-alkoxy-2*H*-chromen-8-amines **10a**–**c** is outlined in Scheme 1. 2-nitro-5-fluorophenol (**1**) reacted with propargyl bromide in dimethylformamide in the presence of potassium carbonate to produce compound **2** [14]. Compound **4** was obtained through the reaction of **2** with sodium 4-methoxyphenylethoxylate (**3**). Claisen thermal rearrangement of **4** in *N*,*N*-diethyl aniline at 180–200 °C led to the production of 5-(4-methoxybenzyloxy)-8-nitro-2*H*-chromene (**5**) [15]. Compound **5** was debenzylated with trifluoroacetic acid in dichloromethane to produce 5-hydroxyl-8-nitro-2*H*-chromene (**6**). 5-(2-Bromoethoxy)-8-nitro-2*H*-chromene (**7)** was obtained by reacting **6** with 1,2-dibromoethane in CH_3_CN under reflux for 24 h. Compound **7** reacted with morpholine (**8a**), *cis*-2,6-dimethylmorpholine (**8b**), and triazole (**8c**) in dimethylformamide (DMF) at 80 °C in the presence of K_2_CO_3_ to yield the corresponding compounds **9a**–**c**, which were then reduced to the 5-alkoxy-2*H*-chromen-8-amines **10a**–**c** with SnCl_2_ in refluxing EtOH.

*Reagents and conditions*: (i) propargyl bromide, K_2_CO_3_/DMF (88%); (ii) NaH/DMF/−35 °C; (iii) 180–200 °C/*N*,*N*-diethyl aniline; (iv) CF_3_COOH/CH_2_Cl_2_, 0 °C; (v) BrCH_2_CH_2_Br,K_2_CO_3_/CH_3_CN, reflux, 24 h; (vi) R_1_H, K_2_CO_3_, DMF, 80 °C; (vii) SnCl_2_·2H_2_O/EtOAc and EtOH.

The synthetic approach to the chroman-8-amine derivatives **17a**–**f** is outlined in Scheme 2. 5-hydroxy-8-nitro-2*H*-chromene (**7**), prepared as described in Scheme 1, was reduced with H_2_NNH_2_/H_2_O in the presence of Pd/C in ethanol to give 5-hydroxy-8-aminochromane (**11**). The amino group of **11** was protected with di-*tert*-butyldicarbonate to produce *N*-Boc-5-hydroxy-8-amino-chromane (**12**). Compound **12** reacted with 1,2-dibromoethane (**13**) through an intramolecular SN_2_ reaction to give 5-(2-bromoethoxy)-8-aminochromane (**14**), which was then substituted with morpholine (**15a**), *cis*-2,6-dimethylmorpholine (**15b**), imidazole (**15c**) and triazole (**15d**) to yield the corresponding compounds **16a**–**d**. Deprotection of **16a**–**d** by treatment with trifluoroacetic acid in dichloromethane produced the 8-aminochromane derivatives **17a**–**f**. The preparation of 5-(2-(pyridin-4-yl)ethoxy)chroman-8-amine (**17e**) was accomplished by a Mitsunobu reaction of *N*-boc-5-hydroxy-8-aminochromane (**12**) and 2-(pyridin-4-yl)ethanol (**18**) with triphenylphosphine (Ph_3_P) and diisopropyl azodicarboxylate (DIAD) in CH_2_Cl_2_ [15] and subsequent cleavage of the Boc group by employing trifluoroacetic acid in CH_2_Cl_2_. Another 8-aminochroman derivative (compound **17f**) was obtained by oxyalkylation of compound **12** with 4-(2-chloroacetyl)morpholine (**20**), which was prepared by the reaction of the morpholine (**15a**) and chloroacetyl chloride (**19**) in the presence of triethylamine, followed by cleavage of the Boc group.

*Reagents and conditions*: (viii) H_2_NNH_2_/H_2_O, Pd/C, EtOH; (ix) (Boc)_2_O, TEA/CH_2_Cl_2_; (x) K_2_CO_3_/CH_3_CN, heat; (xi) K_2_CO_3_/DMF, heat; (xii) trifluoroacetic acid/CH_2_Cl_2_; (xiii) Ph_3_P, DIAD/CH_2_Cl_2_; (xiv) chloroacetyl chloride, TEA/CH_2_Cl_2_; (xv) K_2_CO_3_/DMF, heat.

The preparation of 1-aryl-3-*tert*-butyl-*1*H-pyrazol-5-amines **25a**–**g**, **25l** was conducted according to the procedures of Regan *et al.* (Scheme 3) [16] Diazotization of arylamine **21** with NaNO_2_, followed by reduction with SnCl_2_ in HCl at 0 °C, led to the corresponding arylhydrazine hydrochlorides **23a**–**j** [17], which were condensed with pivaloylacetonitrile (**24**) in ethanolic solution of HCl at reflux to give **25a**–**g** and **25l** [18]. The other four pyrazol-5-amines **25k**–**n** are commercially available. The structures of **25a**–**l** are listed in Table 1.

*Reagents and conditions*: (xvi) (1) NaNO_2_/H^+^, −5 °C; (2) SnCl_2_, H^+^, −5 °C; (xvii) HCl, EtOH, reflux.

*Reagents and conditions*: (xviii) chloroacetyl chloride/AlCl_3_/toluene; (xix) phthalimide potassium salt, DMF, 70 °C; (xx) *N*,*N*-dimethylformamide dimethyl acetal/DMF, 100 °C; (xxi) *tert*-butyl hydrazine/90% ethanol, reflux; (xxii) 85% H_2_NNH_2_/H_2_O/ethanol; (xxiii) NaHCO_3_/DMF, 75 °C; (xxiv) NaOH/H_2_O, 50 °C; (xxv) H_2_O/50 °C at pH 10–11.

**Table 1:** 1-Aryl-3-tert-butyl-*1*H-pyrazol-5-amines **25a**–**l**. 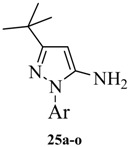

Compound	Ar	Compound	Ar	Compound	Ar	Compound	Ar
**25a**	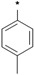	**25b**	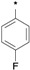	**25c**	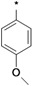	**25d**	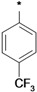
**25e**	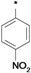	**25f**	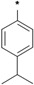	**25g**	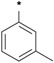	**25h**	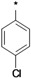
**25i**	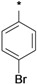	**25j**	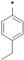	**25k**	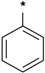	**25l**	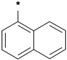

The other four arylamines (compounds **25m**, **25n**, **25o**, **25p)** were prepared as shown in Scheme 4. 1-*tert*-Butyl-3-*p*-tolyl-1*H*-pyrazol-4-amine (**25m**) was prepared by referring to similar reaction conditions [19]. 2-Chloro-1-*p*-tolylethanone (**27**) was obtained by Friedel-Crafts acylation of toluene with chloroacetyl chloride. Condensation of **27** with phthalimide potassium salt (**28**) in DMF gave a good yield of 2-(1,3-dioxoisoindolin-2-yl)-1-*p*-tolylethanone (**29**). Reaction of **29** with dimethyl-formamide dimethyl acetal (**30**) at reflux gave the enamine **31** in quantitative yield. Reaction of **31** with hydrazine in ethanol at room temperature for one hour gave 4-aminopyrazole (**25m** 78%. 4-*tert*-butyl-1-*p*-tolyl-*1*H-imidazol-2-amine (**25n**) was obtained referring the method described in literature [20]. N-(pivaloylmethyl)-4-methylaniline (**36**), which was synthesized from 4-methylaniline (**34**) and α-chloropinacolone (**35**) in DMF using sodium bicarbonate as a deacidifying reagent at 75 °C for 48 h, condensed with cyanamide (**37**) upon heating in ethanol at reflux for 12 h to give **25n** at a yield of 77%. 3-*tert*-Butylisoxazol-5-amine (**25o**) was prepared in good yield (88%) by condensation of pivaloylacetonitrile (**37**) and hydroxylamine hydrochloride (**38**) in alkaline aqueous sodium hydroxide solution at 50 °C [21]. 5-*tert*-Butylisoxazol-3-amine (**25o**), the positional isomer of **25p**, was prepared following the method described in [16]. Thus, pivaloylacetonitrile (**24**) reacted with hydroxylamine hydrochloride (**35**) at *p*H 10–11 and 50 °C for 10 h and then at *p*H 4–5 and 50 °C for 3 h to produce **25p** at a yield of 77%.

Finally, the target *N*-aryl-*N'*-chromenylurea and *N*-aryl-*N'*-chromanylurea derivatives were prepared as shown in Scheme 5. The 5-alkoxy-2*H*-chromen-8-amines **10a**–**c** or the 5-alkoxychroman-8-amines **17a**–**f** reacted with triphosgene in dichloromethane at −15 °C to form the corresponding isocyanate compounds [22], which then reacted with aryl amines **25a**–**p** in the presence of triethylamine at room temperature for 12–24 h to yield the target compounds **39a**–**j** and **40a**–**i**. The structures of **39a**–**j** and **40a**–**i** are listed in Table 1.

*Reagents and conditions*: (xxvi) triphosgene/CH_2_C1_2_, −10–0 °C; (xxvii) Et_3_N/CH_2_C1_2_, r.t. 2–3 days, 15%–40%.

The potential inhibitors **39a**–**j** and **40a**–**r** were profiled for their ability to inhibit TNF-α release in lipopolysaccharide (LPS)-stimulated THP-1 cells [18], with BIRB796 as positive control. The results are shown in Table 2 and Table 3. Among our potential inhibitors, the 2*H*-chromen-5-ylurea compounds **40a**, **40k**, **40l** and **40m** exhibited approximately an order of magnitude more potent anti-TNF-α activity than their corresponding chroman-5-ylurea analogues **39a**, **39c**, **39d** and **39e**. Compounds **39a** and **40a** were identical to BIRB796, except the 2*H*-chromen-5-ylurea ring in BIRB796 was changed to a 2*H*-chromen and chroman moiety, respectively. The TNF-α inhibitory activities decreased in sequence of BIRB796 (IC_50_ = 0.032 μM), **40a** (IC_50_ = 0.050 μM) and **39a** (IC_50_ = 0.31 μM), which is consistent with the downward tendency of the length of the conjugated system of the naphthyl, 2*H*-chromen and chroman rings. We presume that the conjugated system length of the naphthyl ring, 2*H*-chromen and chroman is related to the strength of the π-cation interaction between the conjugated system and the cationic amino groups in the side chains of Lys53, which in turn affects the anti-TNF-α activity of the corresponding derivatives. The activity of 2*H*-chromen-5-yl urea compounds was very close to that of the the corresponding naphthyl urea analogues, which indicates that 3-(2*H*-chromen-5-yl)ureas may serve as a novel chemotype for the development of *p*38MAPKα inhibitors.

When the left side of our molecules utilized the 1-aryl-3-*tert*-butyl-*1*H-pyrazol-5-amine group from BIRB796 (compounds **39f**–**j** and compounds **40q**–**r**), an obvious decrease in TNF-α inhibition activity was seen when the morpholine group was replaced with 1,2,4-triazole (**39h**, **40r**) or pyridine (**39j**). In contrast, a relatively small decrease in activity was observed with the replacement of the morpholine group with 2,6-dimethylmorpholine (**39g**, **40q**) and 1,3-imidazole (**39i**). These results, taken together, indicate that the TNF-α inhibitory activity of our compounds was very sensitive to substitutions in this region. Aromatic hydrogen bond accepting moieties such as 1,2,4-triazole, pyridine and 1,3-imidazole are inferior to the aliphatic morpholine. Introducing two methyl groups to the position *ortho* to the oxygen atom of morpholine is also not conducive to TNF-α inhibitory activity. In our molecules, we also attempted to modify the ethoxy linker of the morpholinoethoxy group with a 2-oxoethoxy. Unfortunately, the resultant compound **39f** exhibited little activity against TNF-α release, which may indicate that a hydrophobic linkage is more suitable than a hydrophilic one for this region.

**Table 2:** Structure and TNF-α inhibitory activity of 1-aryl-3-(chroman-5-yl)urea compounds **40a**–**i**. 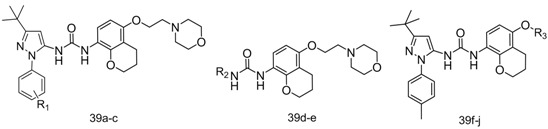

Compound	R_1_	R_2_	R_3_	IC_50_ (μM)
**39a**	4-Methyl			0.31
**39b**	4-H			1.17
**39c**	3-Methyl			0.895
**39d**		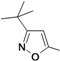		2.0
**39e**		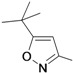		2.5
**39f**			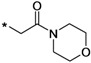	>10
**39g**			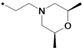	0.50
**39h**			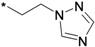	>10
**39i**			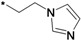	0.48
**39j**			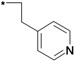	>10
BIRB796				0.032

When the right side of our molecules utilized the 2-morpholinoethoxy group from BIRB796 (compounds **39a**–**e** and **40a**–**p**), changing the 1-(*p*-tolyl)-3-*tert*-butyl-1*H*-pyrazol-5-amine group (compounds **40a**, **39a**) on the left side to 5-*tert*-butylisoxazol-3-amine (compounds **40l**, **39g**) or 3-*tert*-butylisoxazol-5-amine (compounds **40m**, **39h**) led to an apparent decrease in potency. Likewise, in the 2*H*-chromenylurea series, when the 1-(*p*-tolyl)-3-*tert*-butyl-1*H*-pyrazol-5-amine group of **40a** was replaced with its positional isomer 4-*tert*-butyl-1-(*p*-tolyl)-1*H*-imidazol-2-amine (**40n**) or 1-*tert*-butyl-3-(*p*-tolyl)-1*H*-pyrazol-4-amine (**40p**), a dramatic loss of activity was observed. These results demonstrate that the *p*-tolyl group is important to maintain TNF-α inhibitory activity, and the electronic configuration model of the pyrazole ring also significantly affects the activity. To further probe the SAR in this particular region, we also attempted to replace the methyl group of the *p*-tolyl group with other substitution. As seen in Table 3, replacing the 4-methyl group with a larger substituent, such as 4-chloro (**40c**), 4-bromo (**40d**), 4-methoxy (**40e**), 4-trifluoromethyl (**40f**), 4-ethyl (**40h**), 4-isopropyl (**40i**), or 3-chloro-4-fluoro (**40j**) resulted in almost complete disappearance of the TNF-α inhibitory activity (IC_50_ < 10 μM). Changing the *p*-tolyl group for a naphthyl group also abolished the activity (IC_50_ < 10 μM). In contrast, changing the 4-methyl group to a small polar substituent such as 4-fluoro (**40b**) was relatively tolerated and resulted in 4-fold loss of activity, while changing the 4-methyl group to a powererfully polar nitro group provided a potent *p*38MAPKα inhibitor (**40g**) with an IC_50_ value of 0.033 μM, which is comparable to that of BIRB796 (0.032 μM) and higher than that of compound **40a** (0.050 μM).

**Table 3:** Structure and TNF-α inhibitory activity of 1-aryl-3-(2*H*-chromen-5-yl) urea compounds **39a**–**r**. 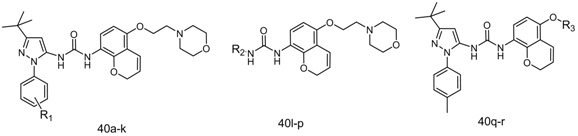

Compound	R_1_	R_2_	R_3_	IC_50_ (μM)
**40a**	4-Methyl			0.050
**40b**	4-Fluoro			0.223
**40c**	4-Chloro			>10
**40d**	4-Bromo			>10
**40e**	4-Methoxy			>10
**40f**	4-Trifluoromethyl			>10
**40g**	4-Nitro			0.033
**40h**	4-Ethyl			>10
**40i**	4-Isopropyl			>10
**40j**	3-Chloro-4-fluoro			>10
**40k**	3-Methyl			0.058
**40l**		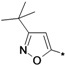		0.25
**40m**		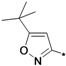		0.42
**40n**		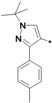		>10
**40o**		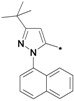		>10
**40p**		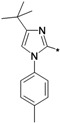		>10
**40q**			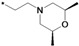	0.16
**40r**			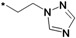	>10
BIRB796				0.032

These results indicate that the TNF-α inhibitory activity of our compounds was also sensitive to the substitutions of the phenyl ring, and 4-nitrophenyl was at least as effective as the 4-methylphenyl group.

## 3. Experimental

### 3.1. General Information

All the reagents were commercially available and used without further purification. ^1^H-NMR spectra were measured using a Bruker-400 (Bruker Company, karlsruhe, Germany) or YS-300 instrument. Mass spectra were obtained from VG300, ZAD-2F or API3000 instruments.

### 3.2. Chemistry

*4-Fluoro-1-nitro-2-(prop-2-ynyloxy) benzene* (**2**). To a stirred solution of 5-fluoro-2-nitrophenol (30 g, 191 mmol) in DMF (300 mL) anhydrous K_2_CO_3_ (52.83 g, 382 mmol) and 3-bromopropyne (27.27 g, 229 mmol) were successively added The mixture was stirred at room temperature for 5 h and then poured into ice water (1,500 mL). A buff precipitate separated out upon standing overnight. The solid was collected by filtration and air-dried to give compound **2** as a buff solid (33.91 g, yield: 85%), mp: 50–52 °C, ^1^H-NMR (CDCl_3_, 400 MHz), *δ*: 7.98 (m, 1H), 6.98 (m, 1H), 6.80 (m, 1H), 4.86 (s, 2H), 2.66 (s, 1H). FAB-MS (*m/z*): 196 [M+H]^+^.

*4-(4-Methoxybenzyloxy)-1-nitro-2-(prop-2-ynyloxy)benzene* (**4**). To a stirred solution of (4-methoxyphenyl)methanol (22.63 g, 163.8 mmol) in anhydrous DMF (200 mL), 60% sodium hydride (8.74 g, 218.4 mmol) was added in portions. The mixture was stirred at ambient temperature until no further gas was released, then cooled to −35 °C. To this solution, compound **2** (21.84 g, 109.2 mmol) was added in one batch. The reaction mixture was stirred for a further 6 h at −35 °C under nitrogen, and then was poured into ice water (1,000 mL). The precipitate was collected, washed with water and air-dried to give compound **4** as a buff solid (27.82 g, yield: 93%), mp: 53–54 °C, ^1^H-NMR (CDCl_3_, 400 MHz), *δ*: 8.03 (d, *J* = 9.24 Hz, 1H), 7.36 (2H, d, *J* = 8.44 Hz), 6.94 (2H, d, *J* = 8.44 Hz), 6.79 (1H, d, *J* = 2.52 Hz), 6.66 (dd, *J* = 9.24 Hz, 2.52 Hz, 1H), 5.08 (2H, s), 4.82 (2H, d, *J* = 2.24 Hz), 384 (3H, s), 2.59 (1H, t, *J* = 2.52 Hz).

*5-(4-Methoxybenzyloxy)-8-nitro-2H-chromene* (**5**). Compound **4** (22 g, 71.8 mmol) was dissolved in *N,N*-diethylaniline (330 mL). The reaction mixture was heated to 195 °C and kept at this temperature for 1 h. After cooling to room temperature, the solvent was distilled off under reduced pressure. The residue was purified by column chromatography on silica gel with ethyl acetate/petroleum ether (3:2) as eluent to give compound **5** as a yellow solid (6.47 g, yield: 29%). ^1^H-NMR (CDCl_3_, 400 MHz), *δ*: 7.85 (1H, d, *J* = 9.52 Hz), 7.32 (2H, d, *J* = 5.64 Hz), 6.94 (2H, d, *J* = 5.64 Hz), 6.79 (1H, dt, *J* = 10.36 Hz, 1.68 Hz), 6.55 (1H, d, *J* = 9.52 Hz), 5.83 (1H, dt, *J* = 10.36 Hz, 3.64 Hz), 5.08 (2H, s), 4.94 (2H, dd, *J* = 3.64 Hz), 3.81(3H, s). ESI-MS (^+^Q, *m/z*): 314 [M+H]^+^, 336 [M+Na]^+^.

8*-Nitro-2H-chromen-5-ol* (**6**). To a solution of compound **5** (18.2 g, 58 mmol) in dichloromethane (200 mL), trifluoroethylacetic acid (10.0 mL) was added dropwise with stirring at −10 °C. The reaction mixture was stirred at this temperature for 8 h, then quenched by addition of ice water (5 mL). The aqueous solution was then adjusted to pH = 10 with 1 N sodium hydroxide and the two phases were separated. The water phase was extracted twice with dichloromethane. The organic phases were combined and washed with water and brine, dried with Na_2_SO_4_ and the solvent removed under vacuum to yield the crude product, which was purified by column chromatography (dichloromethane/methanol 100:2) to give compound **6** as a yellow solid (7.5 g, yield: 67%). ^1^H-NMR (CDCl_3_, 400 MHz), *δ*: 11.11 (1H, s) 7.73 (d, 1H, *J* = 9.24 Hz), 6.68 (dt, 1H, *J* = 10.08 Hz, 1.96 Hz), 6.53 (1H, d, *J* = 9.24 Hz), 5.92 (1H, dt, *J* = 10.08 Hz, 3.64 Hz), 4.87 (2H, dd, *J* = 3.64 Hz, 1.96 Hz).

*5-(2-Bromoethoxy)-8-nitro-2H-chromene* (**7**). To a stirred solution of compound **6** (5.97 g, 31 mmol) in acetonitrile (150 mL) potassium carbonate (5.13 g, 37 mmol) and 1,2-dibromoethane (23.23 g, 124 mmol) were continuously added. The resulting mixture was heated to reflux for 2.5 h and then concentrated. The residue was partitioned between water (50 mL) and ethyl acetate (50 mL). The organic layer was separated, and the aqueous phase extracted with several additional portions of ethyl acetate. The combined organic phase was washed with brine, dried (MgSO_4_) and concentrated to dryness. The residue was separated by column chromatography on silica gel with ethyl acetate/petroleum ether (1/1) as eluent to give compound **7** as a yellow solid (4.69 g, yield: 34%). ^1^H-NMR (CDCl_3_, 400 MHz), *δ*: 7.84 (d, 1H, *J* = 9.2 Hz), 6.82 (dd, 1H, *J* = 10.8 Hz, 1.7 Hz), 6.41 (1H, d, *J* = 9.52 Hz), 6.80 (1H, dt, *J* = 10.36 Hz, 1.68 Hz), 6.44 (1H, d, *J* = 9.52 Hz), 5.88 (1H, dt, *J* = 10.08 Hz, 3.68 Hz). ESI-MS (^+^Q, *m/z*): 300 [M+H]^+^, 302 [M+H]^+^, 322 [M+Na]^+^, 324 [M+Na]^+^.

*4-(2-(8-Nitro-2H-chromen-5-yloxy)ethyl)morpholine* (**9a**). To a stirred solution of compound **7** (2 g, 6.66 mmol) in DMF (60 mL) potassium carbonate (1.4 g, 10.1 mmol) and morpholine (908 mg, 10.42 mmol) were continuously added. The resulting mixture was heated to 80 °C for 2 h. and then poured into cold water and extracted with ethyl acetate. The organic phase was washed with brine, dried over Na_2_SO_4_, and concentrated to dryness. The residue was separated by column chromatography on silica gel with ethyl acetate/petroleum ether (2/1) as eluent to give compound **9a** as a white solid (1.06 g, yield: 52%). mp: 92–94 °C, ^1^H-NMR (CDCl_3_, 400), *δ*: 7.86 (d, *J* = 9.2Hz, 1H), 6.73 (dt, *J* = 10.0, 2.0 Hz, 1H), 6.47 (d, *J* = 9.2 Hz, 1H), 5.86 (dt, *J* = 10, 3.6 Hz, 1H), 4.95 (m, 2H), 4.22 (m, 2H), 3.75 (m, 4H), 2.86 (m, 2H), 2.61 (m, 4H). FAB-MS(*m/z*): 307 [M+H]^+^.

*cis-2,6-Dimethyl-4-(2-(8-nitro-2H-chromen-5-yloxy)ethyl) morpholine* (**9b**). Compound **9b** was prepared following the method described for compound **9a**, except *cis*-2,6-dimetylmopholine was used instead of morpholine as a reactant. Treating compound **7** (2 g, 6.63 mmol) in this manner to afford the title compound resulted in 1.80 g (81% yield) of compound **9b** as a white solid. ^1^H-NMR (CDCl_3_, 400 MHz), *δ*: 7.85 (1H, d, *J* = 9.24 Hz), 6.74 (1H, dt, *J* = 10.12 Hz, 1.96 Hz), 6.47 (1H, d, *J* = 9.24 Hz), 6.74 (1H, dt, *J* = 10.12 Hz, 3.64 Hz), 4.94 (2H, dd, *J* = 3.64 Hz, 1.96 Hz), 4.19 (2H, m), 3.68 (2H, m), 2.81 (4H, m), 1.92 (2H, m), 1.17 (3H, s), 1.16 (3H, s). ESI-MS (^+^Q, *m/z*): 335 [M+H]^+^, 142.

*1-(2-(8-Nitro-2H-chromen-5-yloxy)ethyl)-1H-1,2,4-triazole* (**9c**). Compound **9c** was prepared following the method described for compound **9a**, except 1*H*-1,2,4-triazole was used instead of morpholine as a reactant. Treating compound **7** (2 g, 6.66 mmol) by this method resulted in 1.11 g (58% yield) of compound **9c** as a white solid. ^1^H-NMR (CDCl_3_, 400 MHz), *δ*: 8.21 (1H, s), 8.00 (1H, s), 7.81 (1H, d, *J* = 9.24 Hz), 6.56 (1H, dt, *J* = 10.08 Hz, 1.96 Hz), 6.42 (1H, d, *J* = 9.52 Hz), 5.87 (1H, dt, *J* = 10.08 Hz, 3.64 Hz), 4.94 (2H, dd, *J* = 3.64 Hz, 1.96 Hz), 4.64 (2H, m), 4.54 (2H, m). ESI-MS (^+^Q, *m/z*): 289 [M+H]^+^, 311 [M+Na]^+^.

*5-Hydroxy-8-aminochromane* (**11**). To a solution of 5-hydroxy-8-nitro-2*H*-chromene (**7**, 5.0 g, 25.9 mmol) in anhydrous ethanol (100 mL) two drops of hydrochloric acid and palladium/carbon (2.0 g, 10%) were added. The mixture was heated to 60 °C, then hydrazine hydrate (10.0 mL, 85%) was added dropwise. After refluxing for 6 h, the mixture was cooled and filtered. The filtrate was dried and concentrated to give 2.12 g (50% yield) of compound **11** as an oily product which was sufficiently pure for use in the next step without further purification. ^1^H-NMR (CDCl_3_, 400 MHz), *δ*: 6.44 (d, 1H, *J* = 8.0 Hz), 6.19 (d, 1H, *J* = 8.4 Hz), 4.18 (t, 2H, *J* = 5.2 Hz), 3.54 (s, 2H), 2.65 (t, 2H, *J* = 6.8 Hz), 2.00 (p, 2H).

*N-Boc-5-hydroxy-8-aminochromane* (**12**). To a stirred solution of crude **11** (2.12 g, 12.8 mmol) and triethylamine (3.0 mL) in dichloromethane (100 mL), a solution of (Boc)_2_O (4.0 g, 18.3 mmol) in dichloromethane (20 mL) was added at 0 °C. The mixture was stirred at room temperature overnight, poured into water, extracted with dichloromethane, dried, and concentrated to dryness. The residue was separated by column chromatography on silica gel with ethyl acetate/petroleum ether (2/1) as eluent to give 2.69 g (79% yield) of compound **12** as a white solid. ^1^H-NMR (CDCl_3_, 400 MHz), *δ*: 7.61 (d, 1H), 6.76 (s, 1H), 6.30 (d, 1H, *J* = 12 Hz), 5.30 (s, 1H), 4.17 (t, 2H, *J* = 6 Hz), 2.65 (t, 2H, *J* = 8 Hz), 1.98 (p, 2H), 1.52 (s, 9H).

*N-Boc-5-(2-bromoethoxy)-8-aminochromane* (**14**). A mixture of compound **12** (2.20 g, 8.3 mmol), anhydrous potassium carbonate (1.38 g, 10 mmol), and 1,2-dibromopropane (6.23 g, 33 mmol) in acetonitrile (50 mL) was heated under reflux for 72 h. The resulting mixture was cooled to room temperature and water was added to the mixture, which was then extracted three times with ethyl acetate. The combined ethyl acetate layers were washed with water, brine, and then dried over Na_2_SO_4_, filtered and concentrated to dryness. The residue was separated by column chromatography on silica gel with ethyl acetate/petroleum ether (1/1) as eluent to give 0.83 g (27% yield) of compound **14** as a white solid. ^1^H-NMR (CDCl_3_, 400 MHz), *δ*: 7.78 (d, 1H), 6.85 (s, 1H), 6.35 (d, 1H, *J* = 8 Hz), 4.27 (t, 2H, *J* = 6 Hz), 4.20 (t, 2H, *J* = 8 Hz), 3.65 (t, 2H, *J* = 6 Hz), 2.73 (t, 2H, *J* = 6 Hz), 2.00 (p, 2H), 1.53 (s, 9H).

*N-Boc-5-(2-morpholinoethoxy)-8-aminochromane* (**16a**). A mixture of compound **14** (1.86 g, 5 mmol), anhydrous potassium carbonate (0.83 g, 6.01 mmol) and morpholine (6 mmol) in DMF (20 mL) was heated to 80 °C for 2 h. The resulting mixture was cooled to room temperature and poured onto cold water, and extracted three times with ethyl acetate. The combined ethyl acetate layers were washed with water, brine, and then dried over Na_2_SO_4_, filtered and concentrated to dryness. The residue was separated by column chromatography on silica gel with ethyl acetate/petroleum ether (2/1) as eluent to give 1.56 g (82.4% yield) of compound **16a** as a white solid. ^1^H-NMR (CDCl_3_, 400 MHz), *δ*: 7.77 (d, 1H), 6.81 (s, 1H), 6.35 (d, 1H, *J* = 9.2 Hz), 4.17 (t, 2H, *J* = 5.6 Hz), 4.08 (t, 2H), 3.74 (t, 2H, *J* = 4.4 Hz), 2.80 (t, 2H), 2.60–2.65 (m, 6H), 1.97 (p, 2H), 1.50 (s, 9H).

*N-Boc-5-(2-cis-2,6-dimethyl)-morpholinoethoxy)-8-aminochromane* (**16b**). Compound **16b** was prepared following the method described for compound **16a**, except *cis*-1,6-dimetylmophiline was used instead of morpholine as a reactant. Treating compound **14** (2 g, 5.37 mmol) in this manner produced 1.71 g (90% yield) of compound **16b** as a white solid. ^1^H-NMR (CDCl_3_, 400 MHz), *δ*: 7.76 (d, 1H), 6.81 (s, 1H), 6.35 (d, 1H, *J* = 8.8 Hz), 4.17 (t, 2H, *J* = 4.8 Hz), 4.07 (t, 2H), 3.70 (m, 2H), 2.77–2.84 (m, 4H), 2.65 (t, 2H, *J* = 6.8 Hz), 1.91–1.98 (m, 4H), 1.50 (s, 9H), 1.17 (d, 3H), 1.15 (d, 3H).

*N-Boc-5-(2-(1H-1,2,4-triazol-1-yl)-ethoxy)-8-aminochromane* (**16c**). Compound **16c** was prepared following the method described for compound **16a**, except 1,2,4-triazole was used instead of morpholine as a reactant. Treating compound **14** (2 g, 5.37 mmol) in this manner produced 1.28 g (66% yield) of compound **16c** as a white solid. ^1^H-NMR (CDCl_3_, 400 MHz), *δ*: 8.23 (s, 1H), 7.97 (s, 1H), 7.77 (d, 1H), 6.81 (s, 1H), 6.31 (d, 1H, *J* = 8.8 Hz), 4.57 (t, 2H, *J* = 5.2 Hz), 4.29 (t, 2H, *J* = 5.2 Hz), 4.15 (t, 2H, *J* = 5.2 Hz), 2.51 (t, 2H, *J* = 6.4 Hz), 1.94 (p, 2H), 1.50 (s, 9H).

*N-Boc-5-(2-(1H-imidazol-1-yl)-ethoxy)-8-aminochromane* (**16d**). Compound **16d** was prepared following the method described for compound **16a**, except imidazole was used instead of morpholine as a reactant. Treating compound **14** (2 g, 5.37 mmol) in this manner produced 1.29 g (67% yield) of compound **16d** as a white solid. ^1^H-NMR (CDCl_3_, 400 MHz), *δ*: 7.76 (d, 1H), 7.66 (s, 1H), 7.08 (s, 1H), 7.02 (s, 1H), 6.82 (s, 1H), 6.30 (d, 1H, *J* = 8.8 Hz), 4.34 (t, 2H), 4.17 (t, 2H), 2.58 (t, 2H, *J* = 6.6 Hz), 1.96 (p, 2H), 1.50 (s, 9H).

*N-Boc-5-(2-(pyridin-4-yl)ethoxy)-8-aminochromane* (**16e**). A solution of diethyl azodicarboxylate (DEAD, 1.05g, 5.0 mmol) in THF (25 mL) was slowly added to a solution of triphenylphosphine (1.57 g, 5.0 mmol), compound **12** (1.59 g, 6 mmol), and 2-(pyridin-4-yl)ethanol (616 mg, 5.0 mmol) in CH_2_Cl_2_ (25 mL), and the resulting cloudy mixture was stirred at room temperature for 3 h. After filtration of the mixture, the filtrate was concentrated *in vacuo*. Flash chromatography of the crude product (ethyl acetate/petroleum ether(1/1) afforded 1.76 g (95% yield) of compound **16e** as a colourless oil that gradually crystallized upon standing at room temperature. ^1^H-NMR (CDCl_3_, 400 MHz), *δ*: 8.45 (d, 2H, *J* = 6.0 Hz), 7.80 (d, 1H), 6.87 (d, 2H, *J* = 5.2 Hz), 6.86 (s, 1H), 6.39 (d, 2H, *J* = 8.8 Hz), 4.36 (t, 2H), 4.30 (t, 2H), 4.17 (t, 2H), 2.62 (t, 2H, *J* = 6.4 Hz), 1.94 (p, 2H), 1.51 (s, 9H).

#### 3.2.1. *N*-Boc-5-(2-morpholino-2-oxoethoxy)-8-aminochromane (**16f**)

Step 1: preparation of *4-(2-chloroacetyl)morpholine* (**20**). To a stirred solution of morpholine (17.4 g, 200 mmol) and triethylamine (24.24 g, 220 mmol) in CH_2_Cl_2_ (200 mL), acetyl chloride (24.00 g, 210 mmol) in CH_2_Cl_2_ (volume) was added dropwise. The resulting reaction mixture was stirred at this temperature for a further 4 h, then poured into water, and the aqueous layer extracted twice with methylene chloride. The organic chloride phase was combined, washed with dilute hydrochloric acid and water, and dried over Na_2_SO_4_, and evaporated to dryness to give compound **20** (22.66 g, 69.2% yield) as a pale yellow oil.

Step 2: preparation of *N-boc-5-(2-morpholino-2-oxoethoxy)-8-aminochromane* (**16f**). A solution of compound **12** (1.86 g, 5.0 mmol), anhydrous potassium carbonate (0.83 g, 6.0 mmol) and 4-(2-chloroacetyl)-morpholine (**20**, 982 mg, 6.0 mmol) in DMF (20 mL) was heated to 80 °C for 2 h. The resulting mixture was cooled to room temperature, poured onto cold water and extracted three times with ethyl acetate. The combined ethyl acetate layers were washed with water, brine, and then dried over Na_2_SO_4_, filtrated and concentrated to dryness. The residue was separated by column chromatography on silica gel with ethyl acetate/petroleum ether (1/1) as eluent to give 1.35 g (69% yield) of compound **16f** as a white solid. ^1^H-NMR (CDCl_3_, 400 MHz), *δ*: 7.77 (d, 1H), 6.89 (s, 1H), 6.35 (d, 1H, *J* = 8.8 Hz), 4.63 (s, 2H), 4.19 (t, 2H), 3.60–3.68 (m, 8H), 2.68 (t, 2H, *J* = 6.0 Hz), 1.99 (p, 2H), 1.50 (s, 9H).

Step 3: preparation of *5-(morpholinoethoxy)-8-aminochromane* (**17a**). To a solution of compound **16a** (1.48 g, 4.0 mmol) in CH_2_Cl_2_ (40 mL), precooled trifluoroacetic acid (4.0 mL) was added at 0–4 °C, and the reaction was stirred at this temperature for 5 h. After evaporation, water was added to the residue, and the pH of the mixture was adjusted to 10 by addition of 1 M aqueous NaOH solution. The aqueous layer was extracted with ethyl acetate, washed with water, dried over anhydrous sodium sulfate, filtered, and concentrated to give 930 mg (86% yield) of compound **17a** as a grey solid. This product is unstable and was therefore used without delay for the next step.

*5-(2-(2,6-Dimethyl)-morpholinoethoxy)-8-aminochromane* (**17b**). Compound **17b** was prepared following the method described for compound **17a** employing **16b** (1.64 g, 4.0 mmol) as a reactant, producing compound **17b** as a pale white solid (906 mg, 74% yield). This product is unstable and was therefore used without delay for the next step.

*5-(2-(1,2,4-Triazole)-ethoxy)-8-aminochromane* (**17c**). Compound **17c** was prepared following the method described for the compound **17a** employing **16c** (1.23 g, 4.0 mmol) as reactant, to produce compound **17c** as a white solid (729 mg, 70% yield). This product is unstable and was therefore used without delay for the next step.

*5-(2-Imidazolylethoxy)-8-aminochromane* (**17d**). Compound **17d** was prepared following the method described for the compound **17a** employing **16d** (1.23 g, 4.0 mmol) as reactant, to produce compound **17d** as a white solid (778 mg, 75% yield). This product is unstable and was therefore used without delay for the next step.

*5-(2-(Pyridin-4-yl)ethoxy)-8-aminechromane* (**17e**). Compound **17e** was prepared following the method described for compound **17a** employing **16e** (1.48 g, 4 mmol) as reactant, to produce compound **17e** as a white solid (789 mg, 73% yield). This product is unstable and was therefore used without delay for the next step.

*5-(2-Morpholino-2-oxoethoxy)-8-aminochroman* (**17f**). Compound **17f** was prepared following the method described for compound **17a** employing **16f** (1.17 g, 4 mmol) as reactant, to afford the compound **17f** as a pale white solid (795 mg, 68% yield). This product is unstable and was therefore used without delay for the next step.

*3-tert-Butyl-1-p-tolyl-1H-pyrazol-5-amine* (**25a**). A solution of 4-tolyllhydrazine hydrochloride (5.20 g, 33 mmol) and pentylacyl acetonitrile (3.75 g, 30 mmol) in 0.4 M ethanolic solution of HCl (100 mL) was heated under reflux during 48 h. After cooling to room temperature, 1M NaOH was added to the mixture until the pH reached 10–11. The mixture was partitioned between water and ethyl acetate. The water phase was extracted twice with dichloromethane. The organic phases were combined and washed with water and brine, then dried with Na_2_SO_4_. Evaporation of the solvent *in vacuo* afforded the crude product, which was subjected to recrystallization from ethyl acetate and petroleum ether to produce compound **25a** as a white solid (5.88 g, 86% yield). ^1^H-NMR (CDCl_3_, 400 MHz), *δ*: 7.40 (d, 2H, *J* = 8.4 Hz), 7.25 (d, 2H, *J* = 3.08 Hz), 5.50 (s, 1H), 3.72 (brs, 2H, NH_2_), 2.37 (s, 3H), 1.32 (s, 9H).

*3-tert-Butyl-1-(4-fluorophenyl)-1H-pyrazol-5-amine* (**25b**). The title compound was prepared according to the method used for **25a** except (4-fluorophenyl)hydrazine hydrochloride was used instead of 4-tolylhydrazine hydrochloride. Yield: 79%. ^1^H-NMR (CDCl_3_, 400 MHz), *δ*: 7.59 (d, 2H), 7.10 (d, 2H), 5.58 (s, 1 H), 3.62 (brs, 2H, NH_2_), 1.32 (s, 9H).

*3-tert-Butyl-1-(4-methoxyphenyl)-1H-pyrazol-5-amine* (**25c**). The title compound was prepared according to the method used for **25a**, except (4-methoxyphenyl)hydrazine hydrochloride was used instead of 4-tolylhydrazine hydrochloride. Yield: 87%. ^1^H-NMR (CDCl_3_, 400 MHz): 7.41 (d, 2H), 6.97 (d, 2H), 5.43 is, 1H), 3.83 (s, 3H), 1.35 (s, 9H).

*3-tert-Butyl-1-(4-trifluoromethylphenyl)-1H-pyrazol-5-amine* (**25d**). The title compound was prepared according to the method used for **25a**, except (4-trifluoromethylphenyl)hydrazine hydrochloride was used instead of 4-tolyllhydrazine hydrochloride. Yield: 79%. ^1^H-NMR (CDCl_3_, 400 MHz), *δ*: 7.77 (d, 2H, *J* = 8.4 Hz), 7.69 (d, 2H, *J* = 8.4 Hz), 5.56 (s, 1H), 3.76 (s, 2H), 1.31 (s, 9H).

*3-tert-Butyl-1-(4-nitrophenyl)-1H-pyrazol-5-amine* (**25e**). The title compound was prepared according to the method used for **25a**, except 4-nitrohydrazine hydrochloride was used instead of 4-tolylhydrazine hydrochloride. Yield: 83%. ^1^H-NMR (DMSO-*d_6_*, 400 MHz), *δ*: 8.28 (d, *J* = 6.9 Hz, 2H), 7.93 (d, *J* = 6.9 Hz, 2H), 5.55 (s, 2H), 5.46 (s, 1H), 1.20 (s, 9H).

*3-tert-Butyl-1-(4-isopropylphenyl)-1H-pyrazol-5-amine* (**25f**). The title compound was prepared according to the method used for **25a**, except (4-isopropylphenyl) hydrazine hydrochloride was used instead of 4-tolylhydrazine hydrochloride. Yield: 81%. ^1^H-NMR (CDCl_3_, 400 MHz), *δ*: 7.44 (d, 2H, *J* = 8.4 Hz), 7.29 (d, 2H, *J* = 8.4 Hz), 5.51 (s, 1H), 3.72 (s, 2H), 2.93 (m, 1H), 1.31 (s, 9H), 1.25 (d, 3H), 1.24 (d, 3H).

*3-tert-Butyl-1-m-tolyl-1H-pyrazol-5-amine* (**25g**). The title compound was prepared according to the method used for **25a**, except *m*-tolylhydrazine hydrochloride was used instead of *p*-tolylhydrazine hydrochloride. Yield: 79%. ^1^H-NMR (CDCl_3_, 400 MHz), *δ*: 7.38 (s, 1H), 7.31–7.33 (m, 2H), 7.13 (m, 1H), 5.51 (s, 1H), 3.73 (bs, 2H), 2.39 (s, 3H), 1.31 (s, 9H).

*3-tert-Butyl-1-(4-chlorophenyl)-1H-pyrazol-5-amine* (**25h**), 3-*tert*-butyl-*1*-(4-bromophenyl)-1*H*-pyrazol-5-amine (**25i**), 3-*tert*-butyl-*1*-(4-ethylphenyl)-1*H*-pyrazol-5-amine (**25j**) and 3-*tert*-butyl-1-phenyl-1*H*-pyrazol-5-amine (**25n**) are all commercially available.

*3-tert-Butyl-1-(naphthalen-1-yl)-1H-pyrazol-5-amine* (**25o**). Compound **25o** was prepared according to the method used for **1**, except (naphthalen-1-yl)hydrazine hydrochloride was used instead of 4-tolylhydrazine hydrochloride. Yield: 82%. ^1^H-NMR (CDCl_3_, 400 MHz), *δ*: 7.89–7.91 (m, 2H), 7.51–7.55 (m, 5H), 5.59 (s, 1H), 3.50 (s, 2H), 1.34 (s, 9H).

#### 3.2.2. 1-*tert*-Butyl-3-(*p*-tolyl)-4-amine-1*H*-pyrazole (**25p**)

Step 1: preparation of *2-chloro-1-p-tolylethanone* (**27**). To a suspension of anhydrous aluminum chloride (5.60 g, 40 mmol) in anhydrous toluene (40 mL), chloroacetyl chloride (4.52 g, 40 mmol) was slowly added dropwise. After the aluminum chloride has dissolved*,* the mixture was heated slowly to 80 °C and held at that temperature for 2 h, then cooled and poured into crushed ice (100 g) containing concentrated hydrochloric acid (10 mL). The aqueous layer was extracted three times with toluene. The combined toluene layers were washed successively with 10% aqueous sodium hydroxide, water and brine, dried over anhydrous sodium sulfate and evaporated under reduced pressure. The residue was purified by crystallization from diethyl ether to give the pure compound **27**(5.6 g, yield: 86%). ^1^H-NMR (CDCl_3_, 400 MHz), *δ*: 7.46 (d, 2H, *J* = 7.5 Hz), 7.01 (d, 2H, *J* = 7.5 Hz), 4.63 (s, 2H), 2.35 (s, 3H).

Step 2: preparation of *2-(1,3-dioxoisoindolin-2-yl)-1-p-tolylethanone* (**29**). To a solution of compound **27** (5.06 g, 30 mol) in DMF (30 mL), phthalimide potassium salt (5.56 g, 30 mol) was added, and the resulting mixture was stirred at 70 °C for 2 h, cooled, and then poured into ice water (300 mL). The precipitated white solid was filtered and dried to give compound **29** (7.37 g, yield: 88%). ^1^H-NMR (400 MHz, CDCl_3_), *δ*: 7.88–7.83 (m, 4H), 7.73–7.69 (m, 2H), 7.27 (d, *J* = 7.9 Hz, 2H), 5.08 (s, 2H), 2.40 (s, 3H).

Step 3: preparation of *N,N-dimethyl-3-(isoindoline-1,3-dione-2yl)-2-p-tolylprop-1-en-1-amine* (**31**). To a solution of compound **29** (5.58 g, 20 mmol) in DMF (40 mL), *N,N*-dimethylformamide dimethyl acetal (5.68 g, 24 mmol) was added dropwise. The mixture was heated to 100 °C and kept at this temperature for 24 h, then cooled, poured into ice water (200 mL) and extracted with ethyl acetate. The combined organic phase was dried over anhydrous sodium sulfate, and evaporated *in vacuo* to provide a residue which was purified by silica gel chromatography using ethyl acetate and petroleum ether (1/3) as eluent to obtain compound **31** as a white solid (4.0 g, yield: 62%).

Step 4: preparation of *1-tert-butyl-3-(p-tolyl)-4-phthalimido-pyrazole* (**33**). A solution of compound **31** (4.0 g, 14.3 mmol) and *tert*-butyl hydrazine (1.39 g, 15.7 mmol) in 90% ethanol (200 mL) was heated under reflux for 12 h. After cooling to room temperature, the resulting white crystalline solid was filtered, washed with anhydrous ether and dried to give compound **33** as a white solid (3.73 g, yield: 73%).

Step 5: preparation of *1-tert-Butyl-3-(p-tolyl)-4-amine-1H-pyrazole* (**25p**). To a solution of compound **33** (3.59 g, 10 mol) in ethanol (100 mL), 85% hydrazine hydrate solution (2.36 g, 40 mmol) was added dropwise. The mixture was heated to reflux for 2 h, cooled to room temperature, and concentrated, and ether (20 mL) was added. The precipitated white solid was filtered, washed with anhydrous ether, purified by silica gel chromatography using ethyl acetate and petroleum ether (1/1) as eluent to give compound **25p** as a white solid (1.89 g, yield 83%). ^1^H-NMR (CDCl_3_, 400 MHz) *δ*: 7.21–7.26 (m, 5H), 2.68 (s, 2H), 2.41 (s, 3H), 1.41 (s, 9H).

#### 3.2.3. 1-(4-Methylphenyl)-2-amino-4-*tert*-butyl-imidazole (**25q**)

Step 1: preparation of *N-(pivaloylmethyl)-4-methylaniline* (**36**). To a solution of *tert*-butylchloromethyl ketone (3.23 g, 0.024 mol) and *p*-toluidine (2.14 g, 0.020 mol) in DMF (20 mL), sodium bicarbonate (2.52 g, 0.030 mol) was added. The mixture was stirred at 75 °C for 48 h, then cooled, and poured into ice water (200 mL). The precipitated white solid was filtered and dried to give compound **36** as a white solid (4.08 g, yield: 99%).

Step 2: preparation of *1-(4-methylphenyl)-2-amino-4-tert-butyl-imidazole* (**25q**). A solution of compound **36** (5.0 g, 20 mmol) and 50% aqueous cyanamide (16.92 g, 20 mmol) in ethanol (200 mL) was refluxed for 12 h and concentrated, water was added to the residue. the mixture was extracted three times with ethyl acetate, then the combined organic layers washed with dilute sodium hydroxide solution, water and saturated brine, and dried. The residue was purified by silica gel chromatography using dichloromethane and methanol (100/1) to obtain a white solid product (2.62 g, yield: 57%). ^1^H-NMR (CDCl_3_, 400 MHz), *δ*: 7.26 (m, 4H), 6.35 (s, 1H), 4.18 (s, 2H), 2.39 (s, 3H), 1.27 (s, 9H).

*3-tert-Butyl-5-aminoisoxazole* (**25r**). To an aqueous solution of sodium hydroxide solution (0.84 g, 21 mmol) in water (10 mL), pivaloylacetonitrile (1.25 g, 10 mmol) and hydroxylamine hydrochloride (0.76 g, 11 mmol) were added. The resulting solution was stirred at 50° C for 3 h. The reaction mixture was cooled and the resultant white crystalline solid was filtered, washed with water and dried to provide compound **25r** as a white crystalline solid (1.23 g, yield 88%). ^1^H-NMR (CDCl_3_, 400 MHz), *δ*: 5.03 (s, 1H), 4.32 (bs, 2H), 1.27 (s, 9H).

*5-(tert-Butyl)-3-aminoisoxazole* (**25s**). To a solution of pivaloylacetonitrile (3 g, 23.97 mmol) in water (20 mL), NaOH (1.06 g, 26.4 mmol) and hydroxylamine hydrochloride (1.83 g, 26.4 mmol) were added continuously with stirring. The resulting solution was stirred for approximately 30 min at room temperature, and the pH adjusted to 10–11 with 1 M NaOH. After stirring for 10 h or more at 50 °C, the mixture was cooled and washed two to three times with carbon tetrachloride. The aqueous layer was acidified with concentrated HC1 until the pH = 4–5, and then further stirred for approximately 3 h at 50 °C. The reaction mixture was cooled to room temperature, and adjusted to pH 12 by adding an aqueous solution of 1 N NaOH. The resulting solid was filtered, washed with distilled water, and dried in air to obtain compound **25s** as a white solid (2.0 g, yield: 70%). ^1^H-NMR (DMSO-*d_6_*, 300 MHz), *δ*: 5.49 (s, 1H), 5.40 (s, 2H), 1.21 (s, 9H).

#### 3.2.4. General Procedure for the Preparation of Chromanylureas (**39a**–**j**) and 2*H*-Chromenylureas (**40a**–**r**)

A solution of compounds **10a**–**d** or compounds **17a**–**d** (1.0 mmol) in dichloromethane (10 mL) was slowly added to a stirred solution of triphosgene (109 mg, 0.36 mmol) in dichloromethane (50 mL) over a period of 30 min using a syringe. After stirring for a further 30 min, a solution of compound **25a**–**r** (0.6 mmol) and triethylamine (0.4 mL, 2.77 mmol) in dichloromethane (10 mL) was added in one portion. The reaction mixture was stirred for 2 h at room temperature. After completion of the reaction, the reaction was poured into water (50 mL) and extracted three times with dichloromethane. The organic layer was washed with water (5 mL), sat. NaCl solution (5 mL), and dried over Na_2_SO_4_. After evaporation of solvent under vacuum, the residue was purified by silica gel chromatography to give the desired chromanylurea or 2*H*-chromenylurea compounds.

*1-(3-tert-Butyl-1-p-tolyl-1H-pyrazol-5-yl)-3-(5-(2-morpholinoethoxy)-chroman-8-yl)urea* (**39a**). Yield: 19%, ^1^H-NMR (CDCl_3_, 400 MHz), *δ*: 7.69 (d, 1H, *J* = 8.4 Hz), 7.29–7.31 (m, 3H), 7.16 (m, 2H), 6.84 (s, 1H), 6.31–6.34 (m, 2H), 4.04–4.07 (m, 4H), 3.72 (t, 4H), 2.80 (t, 2H, *J* = 5.2 Hz), 2.60 (t, 2H), 2.59 (t, 4H), 2.31 (s, 3H), 1.91 (p, 2H), 1.34 (s, 9H). FAB-MS (*m/z*): 534.2 [M+H]^+^.

*1-(3-tert-Butyl-1-phenyl-1H-pyrazol-5-yl)-3-(5-(2-morpholinoethoxy)-chroman-8-yl)urea* (**39b**). White solid, yield: 26%. ^1^H-NMR (CDCl_3_, 400 MHz), *δ*: 7.67 (d, 1H, *J* = 8.4 Hz), 7.32–7.45 (m, 2H), 7.34 (m, 3H), 7.28 (m, 1H), 7.03 (s, 1H), 6.35 (s, 1H), 6.31 (d, 1H, *J* = 10.8 Hz), 4.04–4.06 (m, 4H), 3.70 (t, 4H, *J* = 4.8 Hz), 2.77 (t, 2H, *J* = 5.6 Hz), 2.56–2.61 (m, 6H), 1.90 (p, 2H), 1.34 (s, 9H). FAB-MS (*m/z*): 520.2 [M+H]^+^.

*1-(3-tert-Butyl-1-m-tolyl-1H-pyrazol-5-yl)-3-(5-(2-morpholinoethoxy)-chroman-8-yl)urea* (**39c**). White solid, yield: 18%, ^1^H-NMR (CDCl_3_, 400 MHz), *δ*:7.68 (d, 1H, *J* = 8.4 Hz), 7.21–7.26 (m, 4H), 7.11 (d, 1H), 6.83 (s, 1H), 6.36 (s, 1H), 6.32 (d, 1H, *J* = 8.8 Hz), 4.05–4.08 (m, 4H), 3.73 (t, 4H, *J* = 4.4 Hz), 2.80 (t, 2H, *J* = 5.6 Hz), 2.57–2.62 (m, 6H), 2.32 (s, 3H), 1.91 (p, 2H), 1.35 (s, 9H). FAB-MS (*m/z*): 534.2 [M+H]^+^.

*1-(3-tert-Butylisoxazol-5-yl)-3-(5-(2-morpholinoethoxy)-chroman-8-yl)urea* (**39d**). White solid, yield: 19%, ^1^H-NMR (CDCl_3_, 400 MHz), *δ*: 8.59 (s, 1H), 7.67 (d, 1H, *J* = 8.0 Hz), 6.36 (d, 1H, *J* = 9.2 Hz), 6.12 (s, 1H), 4.08–4.10 (m, 4H), 3.75 (t, 4H, *J* = 4.4 Hz), 2.83 (t, 2H, *J* = 5.6 Hz), 2.60–2.64 (m, 6H), 1.92 (p, 2H), 1.30 (s, 9H). FAB-MS (*m/z*): 445.1 [M+H]^+^.

*1-(5-tert-Butylisoxazol-3-yl)-3-(5-(2-morpholinoethoxy)-chroman-8-yl)urea* (**39e**). White solid, yield: 31%. ^1^H-NMR (CDCl_3_, 400 MHz), *δ*: 8.75 (s, 2H), 7.86 (d, 1H, *J* = 8.8 Hz), 6.37 (d, 1H, *J* = 8.8 Hz), 6.07 (s, 1H), 4.23 (t, 2H, *J* = 4.8 Hz), 4.10 (t. 2H), 3.74 (t, 4H, *J* = 4.4 Hz), 2.82 (t, 2H, *J* = 5.2 Hz), 2.61–2.67 (m, 6H), 1.99 (p, 2H), 1.34 (s, 9H). FAB-MS (*m/z*): 445.1 [M+H]^+^.

*1-(3-tert-Butyl-1-p-tolyl-1H-pyrazol-5-yl)-3-(5-(2-morpholino-2-oxoethoxy)chroman-8-yl)urea* (**39f**). White solid, yield: 16%, ^1^H-NMR (CDCl_3_, 400 MHz), *δ*: 7.75 (d, 1H, *J* = 9.2 Hz), 7.34 (d, 2H, *J* = 8.4 Hz), 7.25 (d, 2H), 6.38 (s, 1H), 6.34 (d, 1H, *J* = 9.2 Hz), 4.64 (s, 2H), 4.11 (t, 2H, *J* = 3.6 Hz), 3.63–3.67 (m, 8H), 2.67 (t, 2H, *J* = 6.4 Hz), 2.37 (s, 3H), 1.95 (p, 2H), 1.36 (s, 9H). FAB-MS (*m/z*): 548.1 [M+H]^+^.

*1-(3-tert-Butyl-1-p-tolyl-1H-pyrazol-5-yl)-3-(5-(2-(sn-2,6-dimethylmorpholino)ethoxy)chroman-8-yl)-urea* (**39g**). White solid, yield: 21%, ^1^H-NMR (CDCl_3_, 400 MHz), *δ*: 7.71 (d, 1H, *J* = 9.2 Hz), 7.31 (d, 2H, *J* = 8.4 Hz), 7.21 (d, 2H), 7.17 (s, 1H), 6.31–6.36 (m, 3H), 4.08–4.10 (m, 4H), 3.71 (m, 2H), 2.80–2.86 (m, 4H), 2.63 (t, 2H, *J* = 6.4 Hz), 2.36 (s, 3H), 1.92–1.95 (m, 4H), 1.35 (s, 9H), 1.17 (d, 3H), 1.15 (d, 3H). FAB-MS (*m/z*): 562.2 [M+H]^+^.

*3-(3-tert-Butyl-1-p-tolyl-1H-pyrazol-5-yl)-1-(5-(2-(1H-1,2,4-triazol-1-yl)ethoxy)chroman-8-yl)urea* (**39h**). White solid, yield: 15%, ^1^H-NMR (CDCl_3_, 400 MHz), *δ*: 8.08 (s 1H), 7.82–7.84 (m, 2H), 7.45 (s, 1H), 7.32 (d, 2H, *J* = 8.4 Hz), 7.17 (d, 2H, *J* = 8.4 Hz), 6.40 (s, 1H), 6.27 (d, 1H, *J* = 9.2 Hz), 4.56 (t, 2H, *J* = 4.8 Hz), 4.23 (t, 2H, *J* = 4.8 Hz), 3.77 (t, 2H), 2.33–2.36 (m, 5H), 1.69 (p, 2H), 1.35 (s, 9H). FAB-MS (*m/z*): 516.3 [M+H]^+^.

*3-(3-tert-Butyl-1-p-tolyl-1H-pyrazol-5-yl)-1-(5-(2-(1H-imidazol-1-yl)ethoxy)chroman-8-yl)urea* (**39i**). White solid, yield: 21%, ^1^H-NMR (CDCl_3_, 400 MHz), *δ*: 7.88 (d, 1H, *J* = 8.8 Hz), 7.73 (s, 1H), 7.53 (s, 1H), 7.32 (d, 2H, *J* = 8.0 Hz), 7.15 (d, 2H, *J* = 8.0 Hz), 6.93 (s, 1H), 6.89 (s, 1H), 6.41 (s, 1H), 6.22 (d, 1H, *J* = 8.8 Hz), 4.32 (t, 2H, *J* = 4.8 Hz), 4.12 (t, 2H, *J* = 5.6 Hz), 3.62 (t, 2H), 2.35 (t, 2H), 2.32 (s, 3H), 1.61 (p, 2H), 1.35 (s, 9H). FAB-MS (*m/z*): 515.2 [M+H]^+^.

*1-(3-tert-Butyl-1-p-tolyl-1H-pyrazol-5-yl)-3-(5-(2-(pyridin-4-yl)ethoxy)chroman-8-yl)urea* (**39j**). White solid, yield: 17%, ^1^H-NMR (CDCl_3_, 400 MHz), *δ*: 7.71 (d, 1H), 7.50–7.53 (m, 2H), 7.31 (d, 2H), 7.21 (d, 2H, *J* = 8.0 Hz), 6.37 (s, 1H), 6.30 (d, 1H, *J* = 8.4 Hz), 6.26 (t, 1H), 4.52 (t, 2H, *J* = 5.2 Hz), 4.28 (t, 2H, *J* = 5.2 Hz), 4.07 (t, 2H, *J* = 5.6 Hz), 2.54 (t, 2H, *J* = 6.4 Hz), 2.35 (s, 3H), 1.91 (p, 2H), 1.36 (s, 9H). FAB-MS (*m/z*): 515.1 [M+H]^+^.

*1-(3-tert-Butyl-1-p-tolyl-1H-pyrazol-5-yl)-3-(5-(2-morpholinoethoxy)-2H-chromen-8-yl)urea* (**40a**). Yield: 27%, white solid. ^1^H-NMR (CDCl_3_, 400 MHz), *δ*: 7.74 (d, 1H, *J* = 8.8 Hz), 7.33 (d, 2H, *J* = 8.4 Hz), 7.21 (d, 2H, *J* = 8.0 Hz), 7.13 (s, 1H), 6.72–6.74 (dd, 1H, *J* = 8.4 Hz, 1.6 Hz), 6.39 (d, 1H, *J* = 9.2 Hz), 6.35 (s, 1H), 6.34 (s, 1H), 5.72 (m, 1H), 4.71 (t, 2H, *J* = 2.0 Hz), 4.11 (t, 2H), 3.74 (t, 4H), 2.82 (t, 2H), 2.60 (t, 4H), 2.36 (s, 3H), 1.35 (s, 9H). FAB-MS (*m/z*): 532.2 [M+H]^+^.

*1-(3-tert-Butyl-1-(4-fluorophenyl)-1H-pyrazol-5-yl)-3-(5-(2-morpholinoethoxy)-2H-chromen-8-yl)-urea* (**40b**). Yield: 22%, white solid, ^1^H-NMR (CDCl_3_, 400 MHz), *δ*: 7.67 (d, 1H, *J* = 8.4 Hz), 7.43 (m, 2H), 7.07-–7.11 (m, 3H), 6.73 (d, 1H, *J* = 10.4 Hz), 6.58 (s, 1H), 6.38 (d, 1H, *J* = 9.2 Hz), 6.35 (s, 1H), 5.73 (m, 1H), 4.71 (t, 2H, *J* = 2.0 Hz), 4.11 (t, 2H, *J* = 5.2 Hz), 3.75 (t, 4H), 2.83 (t, 2H), 2.61 (t, 4H), 1.35 (s, 9H). FAB-MS (*m/z*): 536.2 [M+H]^+^.

*1-(3-tert-Butyl-1-(4-chlorophenyl)-1H-pyrazol-5-yl)-3-(5-(2-morpholinoethoxy)-2H-chromen-8-yl)-urea* (**40c**). Yield: 29%, white solid, ^1^H-NMR (CDCl_3_, 400 MHz), *δ*: 7.65 (d, 1H, *J* = 8.8 Hz), 7.43 (d, 2H), 7.35 (d, 2H, *J* = 8.8 Hz), 7.10 (s, 1H), 6.74 (d, 1H, *J* = 10.0 Hz), 6.68 (s, 1H), 6.36–6.38 (m, 2H), 5.72 (m, 1H), 4.69 (t, 2H, *J* = 2.0 Hz), 4.10 (t, 2H, *J* = 5.2 Hz), 3.74 (t, 4H, *J* = 4.4 Hz), 2.81 (t, 2H, *J* = 4.8 Hz), 2.59 (t, 4H), 1.35 (s, 9H). FAB-MS (*m/z*): 552.2 [M+H]^+^.

*1-(1-(4-Bromophenyl)-3-tert-butyl-1H-pyrazol-5-yl)-3-(5-(2-morpholinoethoxy)-2H-chromen-8-yl)-urea* (**40d**). Yield: 26%, white solid, ^1^H-NMR (CDCl_3_, 400 MHz), *δ*: 7.65 (d, 1H), 7.50 (d, 2H, *J* = 8.8 Hz), 7.36 (d, 2H, *J* = 8.8 Hz), 7.12 (s, 1H), 6.77 (s, 1H), 6.73 (d, 1H, *J* = 10.4 Hz), 6.38 (d, 1H, *J* = 7.2 Hz), 6.36 (s, 1H), 5.72 (m, 1H), 4.69 (t, 2H, *J* = 1.6 Hz), 4.10 (t, 2H, *J* = 1.6 Hz), 3.74 (t, 4H, *J* = 4.4 Hz), 2.82 (t, 2H, *J* = 4.8 Hz), 2.60 (t, 4H), 1.34 (s, 9H). FAB-MS (*m/z*): 598.0 [M+H]^+^.

*1-(3-tert-Butyl-1-(4-methoxyphenyl)-1H-pyrazol-5-yl)-3-(5-(2-morpholinoethoxy)-2H-chromen-8-yl)-urea* (**40e**). Yield: 26%, white solid, ^1^H-NMR (CDCl_3_, 400 MHz), *δ*:7.72 (d, 1H, *J* = 8.8 Hz), 7.33 (d, 2H, *J* = 9.2 Hz), 7.25 (s, 1H), 6.88 (d, 2H, *J* = 9.2 Hz), 6.75 (d, 1H), 6.73 (s, 1H), 6.36 (d, 1H), 6.33 (s, 1H), 5.70 (m, 1H), 4.67 (t, 2H, *J* = 1.6 Hz), 4.10 (t, 2H), 3.77 (s, 3H), 3.74 (t, 4H, *J* = 4.4 Hz), 2.80 (t, 2H, *J* = 5.6 Hz), 2.59 (t, 4H), 1.35 (s, 9H). FAB-MS (*m/z*): 548.1 [M+H]^+^.

*1-(3-tert-Butyl-1-(4-(trifluoromethyl)phenyl)-1H-pyrazol-5-yl)-3-(5-(2-morpholinoethoxy)-2H-chromen-8-yl)urea* (**40f**). Yield: 17%, white solid, ^1^H-NMR (CDCl_3_, 400 MHz), *δ*: 7.65 (m, 5H), 7.07 (s, 1H), 6.76 (s, 1H), 6.73 (d, 1H, *J* = 9.6 Hz), 6.40 (s, 1H), 6.38 (d, 1H, *J* = 8.8 Hz), 5.70 (m, 1H), 4.70 (t, 2H, *J* = 1.6 Hz), 4.10 (t, 2H, *J* = 5.2 Hz), 3.74 (t, 4H, *J* = 4.0 Hz), 2.82 (t, 2H), 2.60 (t, 4H), 1.36 (s, 9H). FAB-MS (*m/z*): 586.1 [M+H]^+^.

*1-(3-tert-Butyl-1-(4-nitrophenyl)-1H-pyrazol-5-yl)-3-(5-(2-morpholinoethoxy)-2H-chromen-8-yl)urea* (**40g**). Yield: 37%, white solid, ^1^H-NMR (CDCl_3_, 400 MHz), *δ*: 8.18 (d, 2H, *J* = 9.2 Hz), 7.74 (d, 2H, *J* = 8.8 Hz), 7.55 (d, 1H), 7.30 (s, 1H), 6.71 (d, 1H, *J* = 10.0 Hz), 6.39 (s, 1H), 6.34 (d, 1H, *J* = 9.2 Hz), 5.70 (m, 1H), 4.68 (t, 2H, *J* = 1.6 Hz), 4.09 (t, 2H, *J* = 6.0 Hz), 3.74 (t, 4H, *J* = 4.4 Hz), 2.81 (t, 2H, *J* = 6.0 Hz), 2.60 (t, 4H), 1.34 (s, 9H). FAB-MS (*m/z*): 563.0 [M+H]^+^.

*1-(3-tert-Butyl-1-(4-ethylphenyl)-1H-pyrazol-5-yl)-3-(5-(2-morpholinoethoxy)-2H-chromen-8-yl)urea* (**40h**). Yield: 27%, white solid, ^1^H-NMR (CDCl_3_, 400 MHz), *δ*: 7.74 (d, 1H, *J* = 8.8 Hz), 7.36 (d, 2H, *J* = 8.4 Hz), 7.25 (d, 2H), 7.14 (s, 1H), 6.73 (d, 1H, *J* = 10.0 Hz), 6.35–6.40 (m, 3H), 5.72 (m, 1H), 4.71 (t, 2H, *J* = 2.0 Hz), 4.12 (t, 2H), 3.75 (t, 4H), 2.84 (t, 2H), 2.62–2.68 (m, 6H), 1.36 (s, 9H), 1.22 (t, 3H). FAB-MS (*m/z*): 546.0 [M+H]^+^.

*1-(3-tert-Butyl-1-(4-isopropylphenyl)-1H-pyrazol-5-yl)-3-(5-(2-morpholinoethoxy)-2H-chromen-8-yl)-urea* (**40i**). Yield: 19%, white solid, ^1^H-NMR (CDCl_3_, 400 MHz), *δ*: 7.74 (d, 1H, *J* = 9.2 Hz), 7.34 (d, 2H), 7.26 (d, 2H), 7.15 (s, 1H), 6.73 (d, 1H, *J* = 10.0 Hz), 6.43 (s, 1H), 6.39 (d, 1H, *J* = 9.2 Hz), 6.35 (s, 1H), 5.72 (m, 1H), 4.71 (t, 2H, *J* = 2.0 Hz), 4.09 (t, 2H, *J* = 5.2 Hz), 3.73 (t, 4H), 2.81 (t, 2H), 2.59 (t, 4H), 1.36 (s, 9H), 1.24 (d, 3H), 1.22 (d, 3H). FAB-MS (*m/z*): 560.1 [M+H]^+^.

*1-(3-tert-Butyl-1-(3-chloro-4-fluorophenyl)-1H-pyrazol-5-yl)-3-(5-(2-morpholinoethoxy)-2H-chromen-8-yl)urea* (**40j**). Yield: 27%, white solid, ^1^H-NMR (CDCl_3_, 400 MHz), *δ*: 7.60 (m, 2H), 7.38 (m, 1H), 7.08–7.14 (m, 3H), 6.72 (d, 1H, *J* = 9.6 Hz), 6.38 (d, 1H, *J* = 8.8 Hz), 6.35 (s, 1H), 5.72 (m, 1H), 4.70 (t, 2H, *J* = 1.6 Hz), 4.10 (t, 2H, *J* = 5.2 Hz), 3.74 (t, 4H, *J* = 4.4 Hz), 2.83 (t, 2H), 2.61(t, 4H), 1.34 (s, 9H). FAB-MS (*m/z*): 570.0 [M+H]^+^.

*1-(3-tert-Butyl-1-m-tolyl-1H-pyrazol-5-yl)-3-(5-(2-morpholinoethoxy)-2H-chromen-8-yl)urea* (**40k**). Yield: 21%, white solid, ^1^H-NMR (CDCl_3_, 400 MHz), *δ*: 7.22 (d, 1H, *J* = 8.8 Hz), 7.26–7.29 (m, 3H), 7.15 (d, 2H), 6.73 (d, 1H, *J* = 10.0 Hz), 6.58 (s, 1H), 6.38 (d, 1H, *J* = 9.6 Hz), 6.36 (s, 1H), 5.72 (m, 1H), 4.71 (t, 2H, *J* = 1.6Hz), 4.12 (t, 2H), 3.76 (t, 4H), 2.84 (t, 2H), 2.63 (t, 4H), 2.36 (s, 3H), 1.36 (s, 9H). FAB-MS (*m/z*): 532.1 [M+H]^+^.

*1-(3-tert-Butylisoxazol-5-yl)-3-(5-(2-morpholinoethoxy)-2H-chromen-8-yl)urea* (**40l**). Yield: 39%, white solid, ^1^H-NMR (CDCl_3_, 400 MHz), *δ*: 9.27 (s, 1H), 7.74 (d, 1H, *J* = 8.8 Hz), 7.40 (s, 1H), 6.67 (d, 1H, *J* = 10.0 Hz), 6.37 (d, 1H, *J* = 9.2 Hz), 6.13 (s, 1H), 5.63 (m, 1H), 4.62 (t, 2H, *J* = 1.6 Hz), 4.10 (t, 2H, *J* = 5.6 Hz), 3.77 (t, 4H, *J* = 4.4 Hz), 2.83 (t, 2H, *J* = 5.2 Hz), 2.69 (t, 4H), 1.30 (s, 9H). FAB-MS (*m/z*): 443.0 [M+H]^+^.

*1-(5-tert-Butylisoxazol-3-yl)-3-(5-(2-morpholinoethoxy)-2H-chromen-8-yl)urea* (**40m**). Yield: 33%, white solid, ^1^H-NMR (CDCl_3_, 400 MHz), *δ*: 8.77 (s, 1H), 8.27 (s, 1H), 7.86 (d, 1H, *J* = 8.8 Hz), 6.75 (d, 1H, *J* = 10.0 Hz), 6.42 (d, 1H, *J* = 9.6 Hz), 6.02 (s, 1H), 5.76 (m, 1H), 4.84 (t, 2H, *J* = 2.0 Hz), 4.12 (t, 2H, *J* = 6.8 Hz), 3.76 (t, 4H), 2.84 (t, 2H), 2.62 (t, 4H), 1.36 (s, 9H). FAB-MS (*m/z*): 443.0 [M+H]^+^.

*1-(1-tert-Butyl-3-p-tolyl-1H-pyrazol-4-yl)-3-(5-(2-morpholinoethoxy)-2H-chromen-8-yl)urea* (**40n**). Yield: 21%, white solid, ^1^H-NMR (CDCl_3_, 400 MHz), *δ*: 7.67–7.70 (m, 2H), 7.18–7.20 (m, 4H), 6.73 (d, 1H, *J* = 10.0 Hz), 6.68 (s, 1H), 6.36 (d, 1H, *J* = 9.2 Hz), 5.72 (m, 1H), 5.49 (s, 1H), 4.71 (t, 2H, *J* = 2.0 Hz), 4.11 (t, 2H), 3.76 (t, 4H), 2.83 (t, 2H), 2.62 (t, 4H), 2.39 (s, 3H), 1.46 (s, 9H). FAB-MS (*m/z*): 532.1 [M+H]^+^.

*1-(3-tert-Butyl-1-(naphthalen-1-yl)-1H-pyrazol-5-yl)-3-(5-(2-morpholinoethoxy)-2H-chromen-8-yl)-urea* (**40o**). Yield: 22%, white solid, ^1^H-NMR (CDCl_3_, 400 MHz), *δ*: 7.88–7.90 (m, 2H), 7.48–7.50 (m, 5H), 7.38 (d, 1H), 6.95 (s, 1H), 6.68 (d, 1H, *J* = 10.0 Hz), 6.48 (s, 1H), 6.24–6.27 (m, 2H), 5.67 (m, 1H), 4.63 (t, 2H, *J* = 1.6 Hz), 4.07 (t, 2H, *J* = 5.2 Hz), 3.75 (t, 4H, *J* = 4.4 Hz), 2.82 (t, 2H), 2.61 (t, 4H), 1.40 (s, 9H). FAB-MS (*m/z*): 568.1 [M+H]^+^.

*1-(4-tert-Butyl-1-p-tolyl-1H-imidazol-2-yl)-3-(5-(2-morpholinoethoxy)-2H-chromen-8-yl)urea* (**40p**). Yield: 27%, white solid, ^1^H-NMR (CDCl_3_, 400 MHz), *δ*: 8.03 (d, 1H, *J* = 9.2 Hz), 7.26–7.30 (m, 5H), 6.78 (d, 1H, *J* = 10.0 Hz), 6.44 (s, 1H), 6.41 (d, 1H, *J* = 9.2 Hz), 5.77 (m, 1H), 4.88 (t, 2H), 4.13 (t, 2H, *J* = 6.8 Hz), 3.76 (t, 4H), 2.84 (t, 2H), 2.62 (t, 4H), 2.42 (s, 3H), 1.34 (s, 9H). FAB-MS (*m/z*): 532.4 [M+H]^+^.

*1-(3-tert-Butyl-1-p-tolyl-1H-pyrazol-5-yl)-3-(5-(2-(cis-2,6-dimethylmorpholino)ethoxy)-2H-chromen-8-yl)urea* (**40q**). Yield: 28%, white solid, ^1^H-NMR (CDCl_3_, 400 MHz), *δ*: 7.74 (d, 1H, *J* = 9.2 Hz), 7.33 (d, 2H, *J* = 8.0 Hz), 7.22 (d, 2H), 7.14 (s, 1H), 6.74 (d, 1H, *J* = 10.0 Hz), 6.45 (s, 1H), 6.39 (d, 1H, *J* = 9.2 Hz), 6.35 (s, 1H), 5.72 (m, 1H), 4.71 (t, 2H, *J* = 1.6 Hz), 4.12 (t, 2H, *J* = 4.0 Hz), 3.74 (m, 2H), 2.83 (m, 4H), 2.36 (s, 3H), 1.94 (m, 2H), 1.36 (s, 9H), 1.17 (d, 3H), 1.16 (d, 3H). FAB-MS (*m/z*): 532.4 [M+H]^+^.

1*-(5-(2-(1H-1,2,4-Triazol-1-yl)ethoxy)-2H-chromen-8-yl)-3-(3-tert-butyl-1-p-tolyl-1H-pyrazol-5-yl)-urea* (**40r**). Yield: 16%, white solid, ^1^H-NMR (CDCl_3_, 400 MHz), *δ*: 8.10 (s, 1H), 7.86 (s, 1H), 7.83 (d, 1H, *J* = 8.0 Hz), 7.38 (s, 1H), 7.32 (d, 2H, *J* = 8.4 Hz), 7.18 (d, 2H), 6.39 (s, 1H), 6.36 (d, 1H), 6.29 (d, 1H, *J* = 9.2 Hz), 5.47 (m, 1H), 4.55 (t, 2H, *J* = 4.8 Hz), 4.44 (t, 2H), 4.28 (t, 2H, *J* = 4.8 Hz), 2.33 (s, 3H), 1.35 (s, 9H). FAB-MS (*m/z*): 514.2 [M+H]^+^.

### 3.3. Pharmacology

THP-1 cells from a human monocytic cell line (obtained from the National Platform of Experimental Cell Resource for Sci-Tech of China, Beijing, China) were suspended in culture medium [RPM1 (Gibco-BRL, Gaithersburg, MD, USA) containing 15% fetal bovine serum and 0.02 mM 2-mercaptoethanol], at a concentration of 2.5 × 10^6^ cells/mL and then plated in a 96-well plate (0.2 mL aliquots in each well). Test compounds were dissolved in DMSO then diluted with the culture medium such that the final DMSO concentration was 0.5%. Aliquots (20 μL) of test solution or medium with DMSO (control) were added to each well. The cells were incubated at 37 °C for 30 min. LPS (Sigma, St. Louis, MO, USA) was added to the wells at a final concentration of 0.5 μg/mL, and cells were incubated for an additional 2 h. At the end of the incubation period, culture supernatants were collected and the amount of TNF-α present was determined using an ELISA assay (R&D Systems, Minneapolis, MN, USA) according to the manufacturer’s instructions. The data were evaluated by nonlinear regression analysis (Origin software, version 7.5; OriginLab Corp., Northampton, MA, USA) using a sigmoidal model, and the IC_50_ value for each compound was calculated from the sigmoidal curve.

## 4. Conclusions

A series of 3-(2*H*-chromen-5-yl)urea and 3-(chroman-5-yl)urea derivatives was designed as potential *p*38MAPKα inhibitors based on knowledge of the crystallographic structure of the *p*38MAPKα/BIRB796 complex. To obtain these target compounds, a simple and efficient synthetic method was developed to construct 5-alkoxy-2*H*-chromen-8-amine and 5-alkoxychroman-8-amine skeletons. The synthesized compounds were evaluated for their inhibitory activity against TNF-α release in LPS-stimulated THP-1 cells. Among our compounds, the 3-(2*H*-chromen-5-yl)urea derivatives exhibited relatively good anti-TNFα activity, with compound **40g** blocking TNF-α release with an IC_50_ value of 0.033 μM, which is equipotent to that of BIRB796 (IC_50_ = 0.032 μM). These results indicate that 3-(2*H*-chromen-5-yl)urea compounds may serve as a novel chemotype for the development of *p*38MAPKα inhibitors.
